# Supplementary material for: A Large Animal Model of Heritable Pulmonary Arterial Hypertension Using Gene-edited BMPR2 Sheep
Source: bioRxiv. 2026 Feb 7:2026.02.06.704456. Preprint. [Version 1] doi: 10.64898/2026.02.06.704456 (PMC12889587; doi:10.64898/2026.02.06.704456)
Supplement: Supplement 1 [file media-1.pdf]

Supplementary Figures and Tables

Supplementary Figure 1. Analysis of 23 sheep blastocysts produced through in vitro production with Lamb #4 sperm and oocytes derived from slaughterhouse-collected wild type (WT) ovaries. (A) Nested PCR targeting *BMPR2* was performed to identify 11/23 (47.8%) embryos with the -49 bp deletion. (B) Embryos with only the WT-sized band were Sanger sequenced to identify the 4/23 (17.4%) embryos (underlined number) with both WT and the 7 bp deletion alleles. Vertical dashed line indicates canonical cut site of Cas9 with single guide RNA 1.

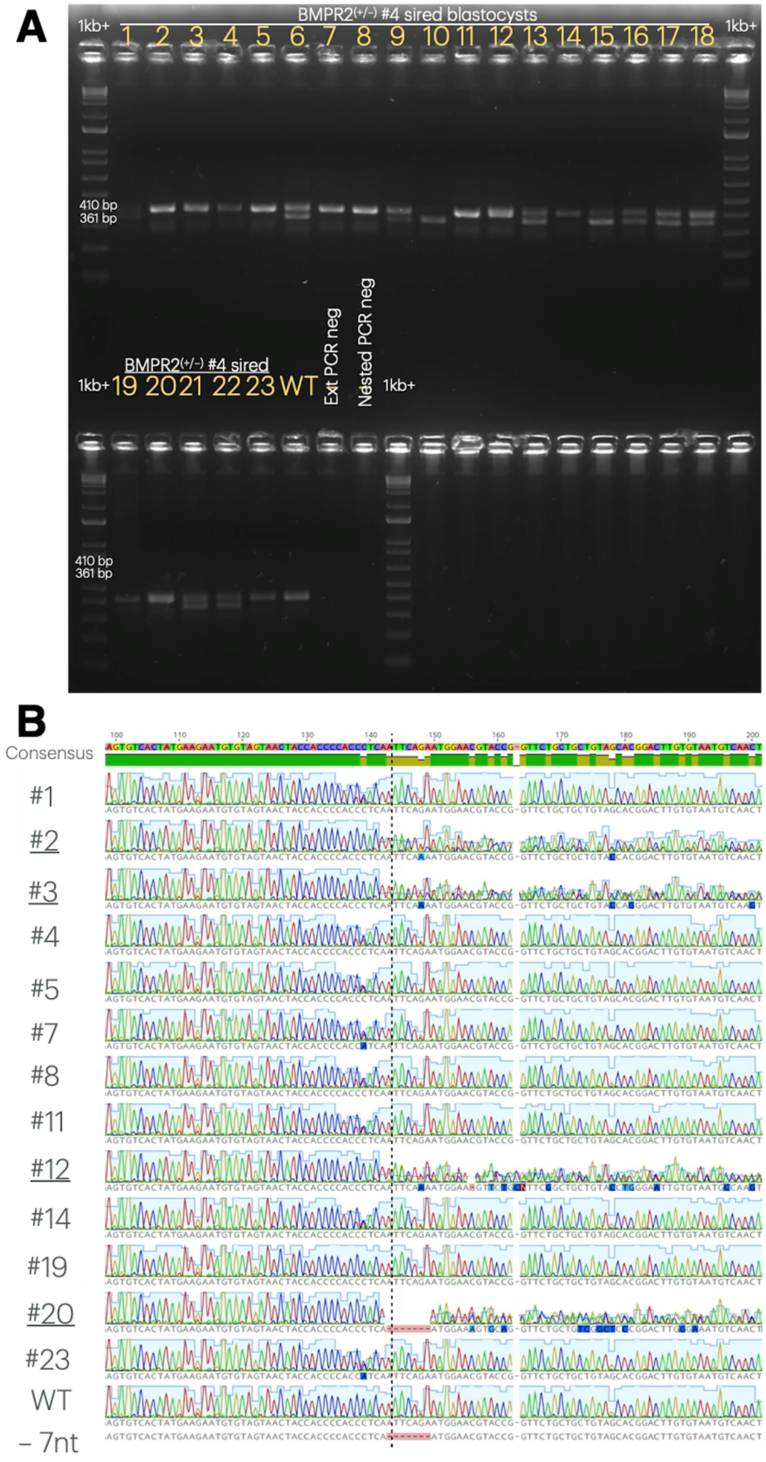

Supplementary Table 1. PCR primers, reaction conditions, cycling conditions, expected wild type (WT) product size and quantitative PCR amplification efficiency (E). Primers were 10 µM except where indicated. GoTaq Green, Promega. KAPAG2, Roche. Q5, NEB. Apex Taq, Genesee Scientific.

Purpose	Primer	Reagents/rxn	Rxn conditions	WT Amplicon Size (bp)	E (%)
Nested_1 for Blastocysts Sanger Sequencing/ TIDER	Intron2-F: ACAGCAGAAGGACTTAGCCA<br>Intron3-R: TCTTGGAAATCCTGGTGTACCTT	1 µL Primer Mix<br>10 µL KAPAG2 Robust ReadyMix<br>4 µL Water<br>5 µL Lysis product	95ºC 5 mins<br>[95ºC 15 s<br>58ºC 30 s<br>72ºC 1 min] x35<br>72ºC 5 mins	838	N/A
Nested_2 for Blastocysts Sanger Sequencing/ TIDER	Intron2-F2: TTGTTTGCAATTTGTTTGTTGTTTTCC<br>Intron3-R2: TCATAATGCTGGAACTCTCTCTCTT	1 µL Primer Mix<br>10 µL KAPAG2 Robust ReadyMix<br>9 µL Water<br>5 µL Nested_1 Product	95ºC 3 mins<br>[95ºC 15 s<br>60ºC 30 s<br>72ºC 1 min] x35<br>72ºC 5 mins	376	N/A
Genotype Tissues/ Blood	Intron2-F2: TTGTTTGCAATTTGTTTGTTGTTTTCC<br>Intron3-R: TCATAATGCTGGAACTCTCTCTCTT	1 µL F Primer<br>1 µL R Primer<br>12.5 µL GoTaq Green<br>10.5 µL Water<br>1 µL DNA	95ºC 5 mins<br>[95ºC 15 s<br>60ºC 30 s<br>72ºC 45 s] x35<br>72ºC 5 mins	376	N/A
Rare/mosaic allele identification (Lambs 1-7)	Intron2-F2: TTGTTTGCAATTTGTTTGTTGTTTTCC<br>Intron3-R: TCTTGGAAATCCTGGTGTACCTT	500 nM ea. primer<br>Q5 High-Fidelity Master Mix	98ºC 30 s<br>[98ºC 10 s<br>60ºC 30 s<br>72ºC 30 s] x35<br>72ºC 5 mins	431	N/A
Rare/mosaic allele identification (Lamb 8)	Exon3-F: GGTGATCCCCAGGAGTGTC<br>7DelIntron3_R: TCTTTGGAGAAAGGAATTTTCAGGA	500 nM ea. primer<br>Q5 High-Fidelity Master Mix	98ºC 2 mins<br>[98ºC 10 s<br>60ºC 30 s<br>72ºC 25 s] x40<br>72ºC 10 mins	201	N/A
Semi-Quantitiative RT-PCR of BMPR2 (Lambs 4, 8)	cDNAEx2_F: CGTCTCTGTGCATTTAAAGATCCA<br>cDNAEx4_R: TGTCAGCATCCTATATCCAAAGCA	400 nM ea primer<br>Apex Taq RED	95ºC 2 mins<br>[95ºC 15 s<br>60ºC 30 s<br>72ºC 45 s] x37<br>72ºC 5 mins	435	N/A
Semi-Quantitiative RT-PCR of ACTB (Lambs 4, 8)	ACTB_F3: GACACCGCAACCAGTTCG<br>ACTB_R3: CCCACCATCACGCCCTG	400 nM ea primer<br>Apex Taq RED	95ºC 2 mins<br>[95ºC 15 s<br>60ºC 30 s<br>72ºC 30 s] x40<br>72ºC 5 mins	157	N/A
Quantitative PCR	ACTB_qPCR_F: TCCTGGGCATGGAATCCTG<br>ACTB qPCR R: GGGGCGCGATGATCTTGAT<br>ACTB Ex5Probe: 6-FAM/CGGGACCAC/ZEN/CATGTACCCTGGCATCG/IABkFQ<br>BMPR2cDNA_F4: TCCCCAGGAGTGTCACTATGAA<br>BMPR2qPCR R3-2: ATGATTATTGTCTCATCTCGGTTAAAT<br>BMPR2_Ex3_WTC_Probe: SUN/CACC+CTC+AAT+TCA+GAATGG/IABkFQ<br>BMPR2_Ex3_WTA_LNA_Probe: SUN/CACCAT+CAA+TTC+AGA+ATGG/IABkFQ	5 µL TaqMan Fast Advanced (ABI)<br>900 nM ea. primer<br>150 nM ea. BMPR2 probe<br>250 nM ACTB probe<br>1 µL cDNA<br>Water to 10 µL final vol.	50ºC 2 mins<br>95ºC 20 s<br>[95ºC 5 s<br>60ºC 20 s] x40	ACTB: 201<br>BMPR2: 189	ACTB: 104<br>BMPR2: 97
Nested_1 for Germline Transmission	Intron2-F: ACAGCAGAAGGACTTAGCCA<br>Intron3-R: TCTTGGAAATCCTGGTGTACCTT	0.4 µL F Primer<br>0.4 µL R Primer<br>10 µL GoTaq Green<br>4.2 µL Water<br>5 µL Lysis product	95ºC 5 mins<br>[95ºC 15 s<br>55ºC 30 s<br>72ºC 90 s] x40<br>72ºC 5 mins		
Nested_2 for Germline Transmission	oBPMR2-Ex3-NestR2_F: TGTACTCCATCACATTGTTTGCA<br>oBPMR2-Ex3-NestR2_R: GCCATTAGCTAGATGTACTCTCA	0.4 µL F Primer<br>0.4 µL R Primer<br>10 µL GoTaq Green<br>7.2 µL Water<br>2 µLNested_1 product	95ºC 2 mins<br>[95ºC 15 s<br>62ºC 30 s<br>72ºC 1 min] x35<br>72ºC 5 mins	410	N/A

Supplementary Table 2. Predicted nuclei acid sequences of edited and wild type (WT) BMPR2 alleles. Snapgene software (www.snapgene.com) predicted the amino acid sequences of BMPR2 edits described in Table 2 and Figure 3. *indicates stop codon, in-frame insertions are bolded, frame-shifted amino acids are underlined, in-frame deletion locations indicated by /.

**WT and WT (CAT)**	MTSSPRRPRRVPSLLWTVLLVSAAAASQNQERLCAFKDPYQQDLGIGESRISHENGTILCSKGSTC<br>YGLWEKSKGDINLVKQGCWSHIGDPQECHYEECVVTTTPPSIQNGTYRFCCCSTDLCNVNFTENFP<br>PPDTTPLSPPHSFNRDETIIIALASVSVLAVLIVALCFGYRMLTGDRKQGLHSMNMMEAAASEPSL<br>DLDNLKLLELIGRGRYGAVYKGSLDERPVAVKVFSXANRQNFINEKNIYRVPLMEHDNIARFIVGD<br>ERVTADGRMEYLLVMEYYPNGSLCKYLSLHTSDWVSSCRLAHSVTRGLAYLHTELPRGDHYKPAIS<br>HRDLNSRNVLVKNDGTCVISDFGLSMKLTGNXLVRPGEEDNAAISEVGTIRYMAPEVLEGAVNLRD<br>CESALKQVDMYALGLIYWEIFMRCTDLFPGXSVPEYQMAFQTEVGNHPTFEDMQVLVSREKQRPKF<br>PEAWKENSLAVRSLKETIEDCWDXDAEARLTAQCAEERMAELMMIWERNKSVSPTVNPMSTAMQNE<br>RNLSHNRRVPKIGPYPDYSSSSYIEDSIHHTDSIVKNISSEHSMSSTPLTIGEKNRNSINYERQQA<br>QARIPSPETSVTSLSTNTTTTNTTGLTPSTGMTTISEVPYPDETSLHATNVSQPVGPTPVCLQLTE<br>EDLETNKLDPKEVDKNLKESSDENLMEHSLKQFSGPDPLSSTSSSLPYPLIKLAVEVTGQQDFTQA<br>ANGQACLIPDVPPTQIYPLPKQQNLPKRPTSLPLNTKNSTKEPRLKFGSKHKSNLKQVETGVAKMN<br>TINAAEPHIVTVTMNGVAGRNHSVNSHTATTQYANGVVPSGQTANTVAHRAQEMLQNQFIGEDTRL<br>NINSSPDEHEPLLRREQQAGHDEGVLDRLVDRRERPLEGGRTNSNNNNSNPCSEQEVPTQGVPSTV<br>ADPGPSKPRRAQRPNSLDLSATNVLDGSSLQLGDSTQDGKSGSGEKIKKRVKTPYSLKRWRPSTWV<br>ISTEPLDCEVNNNGKDRAVHSKSSTTVYLADGGTATTMVSKDIGMNCL*
**#1 Allele 2 (+21)**	MTSSPRRPRRVPSLLWTVLLVSAAAASQNQERLCAFKDPYQQDLGIGESRISHENGTILCSKGSTC<br>YGLWEKSKGDINLVKQGCWSHIGDPQECHYEECVVTTTPPS**SPCTPPS**IQNGTYRFCCCSTDLCNV<br>NFTENFPPPDTTPLSPPHSFNRDETIIIALASVSVLAVLIVALCFGYRMLTGDRKQGLHSMNMMEA<br>AASEPSLDLDNLKLLELIGRGRYGAVYKGSLDERPVAVKVFSXANRQNFINEKNIYRVPLMEHDNI<br>ARFIVGDERVTADGRMEYLLVMEYYPNGSLCKYLSLHTSDWVSSCRLAHSVTRGLAYLHTELPRGD<br>HYKPAISHRDLNSRNVLVKNDGTCVISDFGLSMKLTGNXLVRPGEEDNAAISEVGTIRYMAPEVLE<br>GAVNLRDCESALKQVDMYALGLIYWEIFMRCTDLFPGXSVPEYQMAFQTEVGNHPTFEDMQVLVSR<br>EKQRPKFPEAWKENSLAVRSLKETIEDCWDXDAEARLTAQCAEERMAELMMIWERNKSVSPTVNPM<br>STAMQNERNLSHNRRVPKIGPYPDYSSSSYIEDSIHHTDSIVKNISSEHSMSSTPLTIGEKNRNSI<br>NYERQQAQARIPSPETSVTSLSTNTTTTNTTGLTPSTGMTTISEVPYPDETSLHATNVSQPVGPTP<br>VCLQLTEEDLETNKLDPKEVDKNLKESSDENLMEHSLKQFSGPDPLSSTSSSLPYPLIKLAVEVTG<br>QQDFTQAANGQACLIPDVPPTQIYPLPKQQNLPKRPTSLPLNTKNSTKEPRLKFGSKHKSNLKQVE<br>TGVAKMNTINAAEPHIVTVTMNGVAGRNHSVNSHTATTQYANGVVPSGQTANTVAHRAQEMLQNQF<br>IGEDTRLNINSSPDEHEPLLRREQQAGHDEGVLDRLVDRRERPLEGGRTNSNNNNSNPCSEQEVPT<br>QGVPSTVADPGPSKPRRAQRPNSLDLSATNVLDGSSLQLGDSTQDGKSGSGEKIKKRVKTPYSLKR<br>WRPSTWVISTEPLDCEVNNNGKDRAVHSKSSTTVYLADGGTATTMVSKDIGMNCL*
**#2 Allele 2 (-4,+3)**	MTSSPRRPRRVPSLLWTVLLVSAAAASQNQERLCAFKDPYQQDLGIGESRISHENGTILCSKGSTC<br>YGLWEKSKGDINLVKQGCWSHIGDPQECHYEECVVTTTPPTFRMERTGSAAVARTCVMSTLLRIFH<br>LQTQHHSVHLIHLTEMRQ*
**#3 Allele 3 (+1)**	MTSSPRRPRRVPSLLWTVLLVSAAAASQNQERLCAFKDPYQQDLGIGESRISHENGTILCSKGSTC<br>YGLWEKSKGDINLVKQGCWSHIGDPQECHYEECVVTTTPPSNSEWNVPVLLL*
**#4 Allele 2 (-49)**	MTSSPRRPRRVPSLLWTVLLVSAAAASQNQERLCAFKDPYQQDLGIGESRISHENGTILCSKGSTC<br>YGLWEKSKGDINLVKQGCWSHIGDPQECHYEECVVTTTPPSMSTLLRIFHLQTQHHSVHLIHLTEM<br>RQ*
**#4 Allele 3 (-7) and #6 Allele 2 (-7)**	MTSSPRRPRRVPSLLWTVLLVSAAAASQNQERLCAFKDPYQQDLGIGESRISHENGTILCSKGSTC<br>YGLWEKSKGDINLVKQGCWSHIGDPQECHYEECVVTTTPPSMERTGSAAVARTCVMSTLLRIFHLQ<br>TQHHSVHLIHLTEMRQ*
**#5 Allele 2 (-9,+1)**	MTSSPRRPRRVPSLLWTVLLVSAAAASQNQERLCAFKDPYQQDLGIGESRISHENGTILCSKGSTC<br>YGLWEKSKGDINLVKQGCWSHIGDPQECHYEECVVTTTPPSTNVPVLLL*
**#5 Allele 2 (-15)**	MTSSPRRPRRVPSLLWTVLLVSAAAASQNQERLCAFKDPYQQDLGIGESRISHENGTILCSKGSTC<br>YGLWEKSKGDINLVKQGCWSHIGDPQECHYEECVVT/IQNGTYRFCCCSTDLCNVNFTENFPPPDT<br>TPLSPPHSFNRDETIIIALASVSVLAVLIVALCFGYRMLTGDRKQGLHSMNMMEAAASEPSLDLDN<br>LKLLELIGRGRYGAVYKGSLDERPVAVKVFSXANRQNFINEKNIYRVPLMEHDNIARFIVGDERVT<br>ADGRMEYLLVMEYYPNGSLCKYLSLHTSDWVSSCRLAHSVTRGLAYLHTELPRGDHYKPAISHRDL

	NSRNVLVKNDGTCVISDFGLSMKLTGNXLVRPGEEDNAAISEVGTIRYMAPEVLEGAVNLRDCESA<br>LKQVDMYALGLIYWEIFMRCTDLFPGXSVPEYQMAFQTEVGNHPTFEDMQVLVSREKQRPKFPEAW<br>KENSLAVRSLKETIEDCWDXDAEARLTAQCAEERMAELMMIWERNKSVSPTVNPMSTAMQNERNLS<br>HNRRVPKIGPYPDYSSSSYIEDSIHHTDSIVKNISSEHSMSSTPLTIGEKNRNSINYERQQAQARI<br>PSPETSVTSLSTNTTTTNTTGLTPSTGMTTISEVPYPDETSLHATNVSQPVGPTPVCLQLTEEDLE<br>TNKLDPKEVDKNLKESSDENLMEHSLKQFSGPDPLSSTSSSLPYPLIKLAVEVTGQQDFTQAANGQ<br>ACLIPDVPPTQIYPLPKQQNLPKRPTSLPLNTKNSTKEPRLKFGSKHKSNLKQVETGVAKMNTINA<br>AEPHIVTVTMNGVAGRNHSVNSHTATTQYANGVVPSGQTANTVAHRAQEMLQNQFIGEDTRLNINS<br>SPDEHEPLLRREQQAGHDEGVLDRLVDRRERPLEGGRTNSNNNNSNPCSEQEVPTQGVPSTVADPG<br>PSKPRRAQRPNSLDLSATNVLDGSSLQLGDSTQDGKSGSGEKIKKRVKTPYSLKRWRPSTWVISTE<br>PLDCEVNNNGKDRAVHSKSSTTVYLADGGTATTMVSKDIGMNCL*
**#8 Allele 2 (+94)**	MTSSPRRPRRVPSLLWTVLLVSAAAASQNQERLCAFKDPYQQDLGIGESRISHENGTILCSKGSTC<br>YGLWEKSKGDINLVKQGCWSHIGDPQECHYEECVVTTTPPSIQNGTYRFCCCSTDLCNVNYEEMCS<br>NYHPTINSEWNVPVLLL*
